# Origami Morphing Surfaces with Arrayed Quasi‐Rigid‐Foldable Polyhedrons

**DOI:** 10.1002/advs.202402128

**Published:** 2024-07-31

**Authors:** Jiacong Li, Jiali Bao, Chengyeh Ho, Shuguang Li, Jing Xu

**Affiliations:** ^1^ Department of Mechanical Engineering Tsinghua University Beijing 100084 China; ^2^ Beijing Key Laboratory of Precision/Ultra‐Precision Manufacturing Equipments and Control Tsinghua University Beijing 100084 China; ^3^ State Key Laboratory of Tribology in Advanced Equipment Tsinghua University Beijing 100084 China

**Keywords:** foldable polyhedron, morphing surface, quasi‐rigid‐foldability, soft robot

## Abstract

Artificial morphing surfaces, inspired by the high adaptability of biological tissues, have emerged as a significant area of research in recent years. However, the practical applications of these surfaces, constructed from soft materials, are considerably limited due to their low shear stiffness. Rigid‐foldable cylinders are anisotropic structures that exhibit high adaptability and shear stiffness. Thus, they have the potential to address this issue. However, changes in shape and area at both ends during folding can lead to collisions or gaps on the morphing surface. Here, a quasi‐rigid‐foldable (QRF) rate is first introduced to quantify the rigid‐foldability of a foldable structure and validate it through experiments. More importantly, a QRF polyhedron is then proposed, which is not only notably anisotropic, similar to a rigid‐foldable cylinder, but also exhibits a zero‐Poisson's ratio property, making it suitable for arraying as morphing surfaces without any collisions or gaps. Such surfaces have a myriad of applications, including modulating electromagnetic waves, gripping fragile objects, and serving as soles for climbing robots.

## Graphical abstract

A quasi‐rigid‐foldable (QRF) polyhedron is proposed to be arrayed as morphing surfaces. The QRF polyhedron is not only notably anisotropic, similar to a rigid‐foldable cylinder, but also exhibits zero‐Poisson's ratio property, making it suitable for arraying as morphing surfaces without any collisions or gaps. Such surfaces have myriad applications, including modulating electromagnetic waves, gripping fragile objects, and serving as soles for climbing robots.

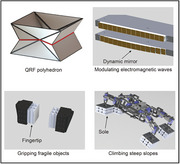

## 1 Introduction

In nature, animals and plants commonly use tissue morphing strategies to interact with the environment.^[^ ^1^, ^2^, ^3^, ^4^, ^5^ ^]^ By mimicking this phenomenon, numerous artificial surfaces have shown promising applications in robotic manipulation,^[^ ^6^, ^7^, ^8^ ^]^ wearable electronics,^[^ ^9^, ^10^ ^]^ biomedical devices,^[^ ^11^, ^12^ ^]^ and various other tasks.^[^ ^13^, ^14^ ^]^ Many of these surfaces are based on arrayed units of simple shaping principles, such as discretizing a 3D shape by its 2D projection and height.^[^ ^15^ ^]^ Compact, motorless, and gearless soft arrays are gaining popularity due to their structural adaptability and straightforward actuation compared to rigid arrays. However, the low shear stiffness of soft materials generally limits their application scenarios.

**Figure 1:**
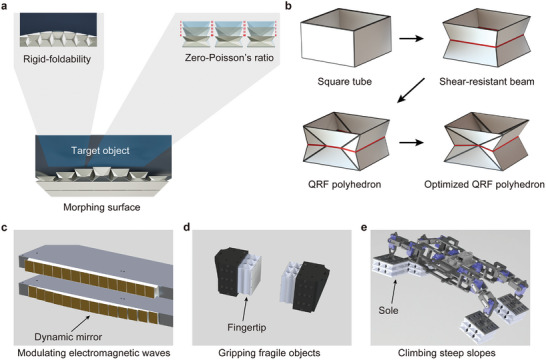
Origami morphing surfaces based on arrayed quasi‐rigid‐foldable polyhedrons with zero‐Poisson's ratio. a) A morphing surface necessitates units with rigid‐foldability and zero‐Poisson's ratio. b) The design and optimization process of the QRF polyhedron. c–e) Schematics of the QRF polyhedron arrayed morphing surface's applications as a dynamic mirror for modulating planar electromagnetic waves, as fingertips to grip fragile objects, and as soles for a climbing robot.

Thanks to their compactness and high shear stiffness, origami‐inspired lightweight skeletal foldable polyhedrons have been recently proposed to address certain issues.^[^ ^15^, ^16^ ^]^ Meanwhile, to adapt to more complex surfaces, it is anticipated that the foldable cylinders will be rigid‐foldable (**Figure** **1**a). For rigid‐foldable cylinders, bending is confined to the folds while the facets remain stationary during the continuous folding process.^[^ ^17^, ^18^ ^]^ This implies rigid‐foldable cylinders possess the lowest theoretical normal stiffness and can be easily modeled and controlled analytically.^[^ ^18^, ^19^, ^20^, ^21^ ^]^The known examples of rigid‐foldable cylinders and cells include the Zipper, zonogon extrusion cell, and Tachi‐Miura Polygon (TMP).^[^ ^22^, ^23^ ^]^

However, rigid‐foldable cylinders lack the zero‐Poisson's ratio property, which is also required by a morphing surface (Figure 1a). A unit without zero‐Poisson's ratio property will lead to variations in shape and area at both ends during folding, causing collisions or gaps on the morphing surface.^[^ ^24^, ^25^, ^26^ ^]^ The collisions will prevent further morphing, while the gaps will reduce shear stiffness for environmental interaction, causing unexpected object manipulation issues.^[^ ^27^ ^]^ Although previous studies have proposed a seamless tessellation of rigid‐foldable cylinders called “cellular structure”, the unit in the array can only move synchronously,^[^ ^24^, ^28^ ^]^ which cannot meet the requirements of the individual unit motion of a morphing surface. Besides, it's important to note that no truly rigid‐foldable cylinder exists in reality, as no material is entirely rigid or has zero thickness.

Consequently, it is imperative to employ units with zero‐Poisson's ratio property and strive for maximum proximity to a rigid‐foldable structure. Here, we define and experimentally validate a quasi‐rigid‐foldable (QRF) rate to quantify the rigid‐foldability of a foldable structure. Previous works have proposed similar concepts to the QRF rate, such as “quasi‐zero‐stiffness”,^[^ ^29^, ^30^ ^]^ “pseudo rigid body”,^[^ ^30^, ^31^, ^32^, ^33^ ^]^ and “pseudo rigid foldable”,^[^ ^33^, ^34^ ^]^ but these studies did not provide a quantitative criterion or explanation for structural flexibility. According to the QRF rate, we optimize a polyhedron with zero‐Poisson's ratio to closely approximate a rigid‐foldable cylinder (Figure 1b).

The optimized QRF polyhedron exhibits high adaptability and exceptional anisotropy. Its normal stiffness is as low as the Zipper, a well‐known rigid‐foldable cylinder, while the shear stiffness is at least 16–38 times higher than the normal stiffness. Moreover, the QRF polyhedron can withstand up to 70% compressive strain of its original height without failure for over 200 load cycles. We demonstrated various applications of morphing surfaces arrayed by the QRF polyhedrons, such as functioning as a dynamic mirror to modulate planar electromagnetic waves (Figure 1c), as fingertips to grip fragile objects (Figure 1d), and as soles for a climbing robot (Figure 1e).

## 2 Results and Discussion

### 2.1 Definition of the QRF Rate and Optimization Framework for Foldable Polyhedrons

**Figure 2:**
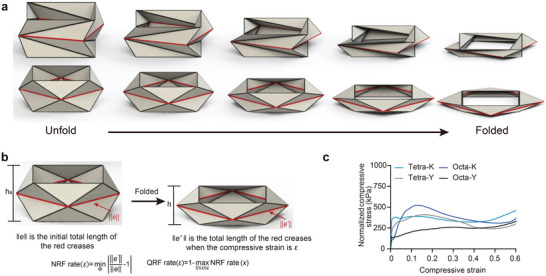
Definition of the QRF rate and optimization framework based on it. a) The assumed continuous folding process of non‐rigid‐foldable Kresling (top) and Yoshimura‐ori (bottom). b) Schematic definition of the NRF rate and QRF rate. c) Representative normalized compressive stress–strain curves of tetragonal Kresling, octagonal Kresling, tetragonal Yoshimura‐ori, and octagonal Yoshimura‐ori demonstrating the rationality of the QRF rate definition.

Previous research has proposed many models to analyze the folding process of a foldable structure,^[^ ^35^, ^36^, ^37^, ^38^, ^39^ ^]^ among which the bar and hinge model is one of the most common models.^[^ ^40^, ^41^, ^42^ ^]^ Inspired by the bar and hinge model, we can consider changes in the creases (stretching and bending) during the folding process to represent the facet deformation.^[^ ^37^ ^]^ Therefore, we attempt to study the mechanics of foldable polyhedrons based on their creases (**Figure** **2**a). If we assume a polyhedron is rigid‐foldable, the length of all creases would remain unchanged while folding.^[^ ^17^, ^18^ ^]^ However, this contradicts the mathematical theorem that the volume of the polyhedron must be constant when the surface area is constant.^[^ ^26^ ^]^ In other words, for a non‐rigid‐foldable (NRF) polyhedron, the length of all creases could change during the continuous folding process. And the greater the total length of the creases changes, the less rigid‐foldable the polyhedron is. However, calculating the change of all creases is very complicated, so we would like to simplify the calculation by only calculating the change of the circumferential creases on the horizontal plane (in red) while assuming the length of other creases remains unchanged.

On the basis of this assumption, we normalize the change ratio of crease length on the horizontal plane by the original length to define the NRF rate (Figure 2b).

(1) $$\varepsilon =\frac{h_{0}-h}{h_{0}}$$

(2) $$NRF\;rate\;\left(\varepsilon \right)=\min_{\Phi }\left|\frac{∥e^{\prime }∥}{∥e∥}-1\right|$$

where *ε* is the compressive strain, *h*_0_ is the initial height, *h* is the height after folding, Φ is the set of all possible folding processes, ‖*e*‖ is the initial total length of the red creases and ‖*e*′‖ is the total length of the red creases after folding. During a folding process, the NRF rate is a function of the compressive strain. The QRF rate can be defined as

(3) $$\mathrm{QRF}\;\mathrm{rate}\left(\varepsilon \right)=1-\max_{0\leq x\leq \varepsilon }\mathrm{NRF}\;\mathrm{rate}\left(x\right)$$

This definition indicates that for a specific foldable polyhedron, the QRF rate is also a function of the compressive strain. Yet under the same compressive strain, the larger the QRF rate is, the more rigid‐foldable the polyhedron is.

To verify the rationality of the QRF rate definition, we conducted compressive experiments on different polyhedrons including tetragonal Yoshimura‐ori, octagonal Yoshimura‐ori, tetragonal Kresling, and octagonal Kresling (Figure 2c; Figure S4, Supporting Information). The Y‐axis presents the normalized compressive stress $\bar{\sigma }$ which was calculated from the compressive stress σ and the NRF rate.

(4) $$\begin{array}{c} \;\bar{\sigma }=\frac{\;\sigma }{\mathrm{NRF}\;\mathrm{rate}}\; \end{array}$$

During the compressive process, all curves align closely with a straight line parallel to the X‐axis, indicating that the NRF rate is proportional to the compressive stress. The larger the NRF rate is, the larger the compressive stress is, and the smaller the normal stiffness is. In addition, the larger the NRF rate is, the smaller the QRF rate is. This suggests that the normal stiffness of a polyhedron is positively correlated with its QRF rate, through which we can quantify the extent to which NRF structures approach a rigid‐foldable one.

As mentioned above, for foldable polyhedrons to be arrayed as a morphing surface, the zero‐Poisson's property is necessary. Therefore, we proposed an optimization framework for polyhedrons with known crease patterns under the constraint of zero‐Poisson's ratio (Figure S5, Supporting Information). The primary goal is to achieve the smallest possible NRF rate by optimizing the coordinates of the polyhedron's vertices for any pattern‐known foldable polyhedron, which is equivalent to maximizing the rigid‐foldability.

### 2.2 Design of the QRF Polyhedron and Comparison with Other Typical Polyhedrons

To create a morphing surface with smooth edges, we chose to design a foldable polyhedron with a quadrilateral cross‐section. Additionally, to prevent buckling caused by the constraint of the circumferential creases during folding, we incorporated parallelogram and triangle patterns into the lateral sides, which is beneficial for improving structural flexibility (Figure 1b; Figure S6, Supporting Information).

**Figure 3:**
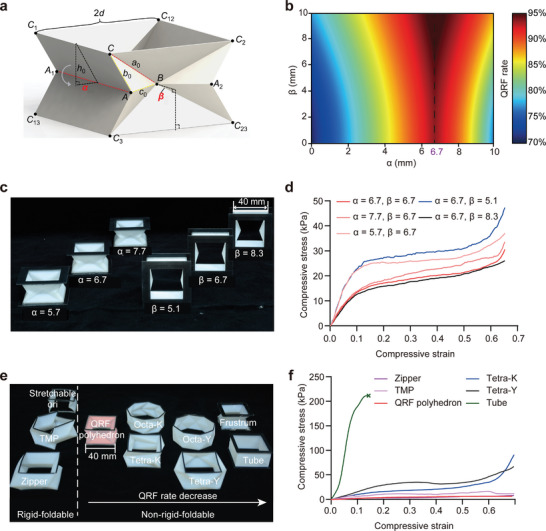
Design of the QRF polyhedron and comparison with other polyhedrons. a) Schematic of the QRF polyhedron and the optimization parameters α and β. b) Heat map showing the QRF rate varying with geometric parameters α and β during continuous folding. The dashed line indicates the optimum α corresponding to the maximum QRF rate. c) Photographs of the QRF polyhedrons with different parameters. d) Representative compressive stress–strain curves of the QRF polyhedrons with different parameters confirming the optimization results. e) Photographs of various foldable polyhedrons arranged by rigid‐foldability. f) Representative compressive stress–strain curves of various foldable polyhedrons showing the notable rigid‐foldability of the QRF polyhedron.

Based on the optimization framework presented above, we introduced and optimized two parameters, the distance between crease AA_1_ and plane CC_1_C_3_ (α), and the distance between vertex B and plane CC_2_C_3_ (β), which represent the degree of concavity on the side of the polyhedron, to further improve the QRF rate of our proposed QRF polyhedron (**Figure** **3**a). In this optimization process, we assumed that the QRF polyhedron is completely compressed, which means that the compressive strain reaches 1. The optimization results show that the QRF rate reaches a local maximum when α = 6.7 but increases monotonically with the increase of β (Figure 3b). Since the QRF rate does not vary significantly with the change of β when α = 6.7, we might also set β = 6.7 for further analysis. To experimentally validate the optimization of the initial polyhedron using the QRF rate, we varied the parameters α and β of the QRF polyhedrons (Figure 3c) and conducted compressive experiments on them. The stress–strain curves of α = 5.7 and 7.7 are significantly higher than those of α = 6.7, and the stress–strain curve of β = 5.1 is higher than that of β = 6.7 and β = 8.3 (Figure 3d), which aligns with our optimization results.

To evaluate the structural flexibility of our proposed QRF polyhedron, we compared it with other foldable polyhedrons including square frustum, tube, tetragonal Yoshimura‐ori, octagonal Yoshimura‐ori, tetragonal Kresling, octagonal Kresling, as well as rigid‐foldable cylinders such as TMP, Zipper, and stretchable ori (Figure 3e). Following the principle of control variables, we kept the heights, radii of circumscribed circles of the top‐end surface, and sidewall thickness of all polyhedrons consistent (Figure S7a, Supporting Information). The QRF polyhedron has the lowest compressive stress at the same compressive strain among the NRF structures compared. Moreover, the curves indicate that the normal stiffness of the QRF polyhedron is extremely close to rigid‐foldable structures like TMP and Zipper (Figure 3f; Figure S7b,c, Supporting Information).

### 2.3 Mechanical Properties of the QRF Polyhedron

**Figure 4:**
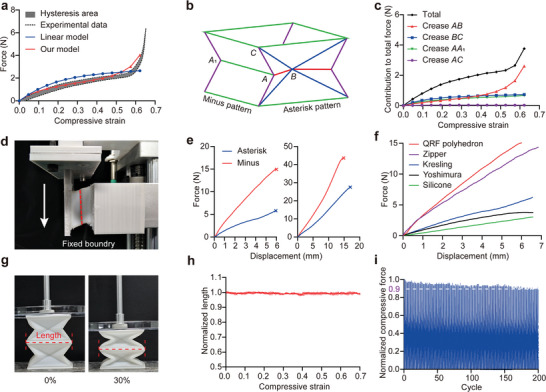
Mechanical properties of the QRF polyhedron. a) Different models of the compressive force‐strain and comparison with the experimental data. b) Schematic of the four types of creases and two kinds of sidewall patterns. c) Contribution to total compressive force of each type of crease. d) Photograph of the shear experimental setup under fixed boundary conditions. e) Shear force‐displacement curves of two kinds of sidewall patterns before (left) and after (right) using Vero White material. f) Shear force‐displacement curves of the QRF polyhedron and four other foldable polyhedrons showing that the QRF polyhedron has the highest shear stiffness. g) Photographs of the QRF polyhedrons when compressive strain is 0% and 30% during a continuous folding process. h) Normalized length‐strain curve of the edge in the middle. N = 3 technical replicates. i) Compressive tests of 200 cycles of the QRF polyhedron under 70% strain.

As previously mentioned, the QRF polyhedron exhibits outstanding structural flexibility. To understand the reason for this, we proposed a model from studying the behavior of each crease (see Note S1, Supporting Information for details). Due to the high QRF rate of the QRF polyhedron, the variations in crease length are minimal and can be neglected. We only consider the width deformation perpendicular to the crease axis caused by the rotation of the facets and use the Gent model to describe the constitutive relationship of soft creases.^[^ ^43^ ^]^ The force‐strain relationship while folding was predicted through the principle of virtual work. Compared to the experimental results and the linear model commonly used for origami structures,^[^ ^19^, ^20^, ^22^ ^]^ our model is more accurate (**Figure** **4**a). As shown in Figure 4b, the creases can be divided into four types: AC, BC, AB, and AA_1_. Our model illustrates the contribution of these four crease types to the total compressive force (Figure 4c). During the initial stage of compression, creases AB, BC, and AA_1_ contribute equally to the total force. However, when the compressive strain exceeds 0.4, the contribution from crease AB begins to increase sharply. Yet, crease AC contributes minimally throughout the whole process due to its small strain.

In addition to its flexibility, we experimentally studied the QRF polyhedron's shear resistance, which is a critical factor for its primary applications in grasping or slope climbing. There are two sidewall patterns of the proposed QRF polyhedron, the asterisk (⁎) pattern and the minus (−) pattern (Figure 4b). We assessed the shear resistance of both sidewalls under fixed boundary conditions (Figure 4d). The maximum shear stress of the minus pattern sidewall before buckling is 15.0 N, with a shear stiffness of 2.862 N·mm^−1^. The asterisk pattern sidewall has a lower shear stiffness of ≈0.946 N·mm^−1^ (Figure 4e), likely due to the more creases in the pattern. The normal stiffness of the QRF polyhedron is only 0.0245–0.0588 N·mm^−1^ (Figure 4c), indicating that the QRF polyhedron is notably anisotropic, even the shear stiffness of the asterisk pattern sidewall is ≈16 – 38 times greater than the normal stiffness. Besides, the shear stiffness can be further enhanced by using Vero White material to construct the four facets forming the crease AA_1_ (Figure 4e). Compared to other foldable cylinders and the silicone block, the QRF polyhedron exhibits the highest shear stiffness (Figure 4f), confirming its superior anisotropy among NRF polyhedrons.

Furthermore, we used a camera to record the length change of the end surface edge during a folding process to verify the zero‐Poisson's ratio property of the QRF polyhedron. To eliminate the friction caused by the experimental equipment, we piled one QRF polyhedron on another and measured the length change of the edge in the middle (Figure 4g). The results indicate that the end surface of the QRF polyhedron remains unchanged during the compressive strain from 0% to 70% (Figure 4h), ensuring that the arrayed morphing surfaces are free of gaps while each unit can deform independently. In terms of durability, when subjected to a 70% cyclic strain, the compressive force only decreased by 10% over 200 cycles (Figure 4i), demonstrating the robustness of the QRF polyhedron under continuous deformation.

### 2.4 Extended Properties of the QRF Polyhedron

**Figure 5:**
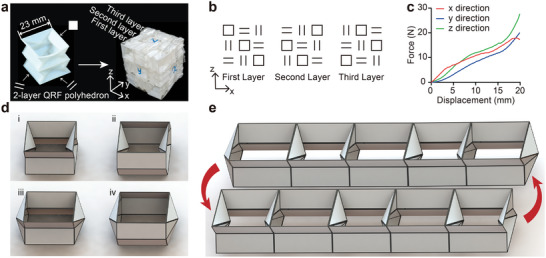
Extended properties of the QRF polyhedron. a,b) Photographs and schematic of how the double‐layer QRF polyhedrons were arrayed as an isotropic cube. c) Force–displacement curves of the isotropic cube in three directions. d) Schematics of four types of folding configurations of the QRF polyhedron. e) The transformation between two steady states of a multistable structure consisting of configurations i and iii.

The QRF polyhedron can be extended to structures with special properties, such as isotropic or multistability. As shown in **Figure** **5**a, 27 units of anisotropic double‐layer QRF polyhedrons can be arranged in a specific pattern to form an isotropic array(Figure 5b; Figure S8, Supporting Information), which exhibits similar compressive force‐displacement curves in all three directions (Figure 5c).

In addition to isotropy, multistability is also a considerable property. The proposed QRF polyhedron can have four types of folding configurations, as long as the material's elasticity and strength allow (Figure 5d). Multipe similar multistable arrayed structures can be achieved according to the way of arrangement, similar to the structure presented in a previous work.^[^ ^44^ ^]^ In Figure 5e, we show the transformation between two steady states of a multistable structure consisting of configurations i and iii. However, as mentioned above, a unit with zero‐Poisson's ratio property is necessary for a morphing surface, so we only use configuration i, the four sidewalls of which are all concave during the folding process, to compose the morphing surfaces.

### 2.5 Applications of the QRF Polyhedron Arrayed Morphing Surfaces

The proposed QRF polyhedron possesses two key properties: zero‐Poisson's ratio and anisotropy. The former allows for the creation of a gap‐free morphing surface, while the latter enables it to withstand large shear forces while maintaining normal flexibility. These two properties enable it to be arrayed as morphing surfaces with a myriad of applications.

**Figure 6:**
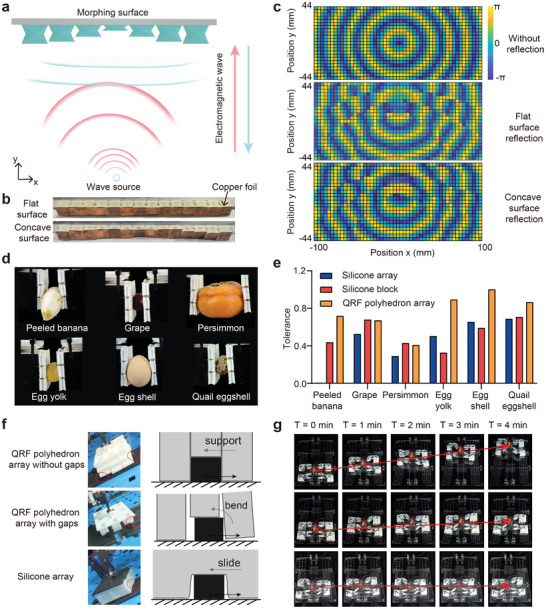
Applications of the QRF polyhedron arrayed morphing surfaces. a) The application of the QRF polyhedron arrayed morphing surface as a dynamic mirror for modulating planar electromagnetic waves. b) Photographs of the QRF polyhedron arrayed dynamic mirror before morphing (top) and after morphing (bottom). c) Phase diagram of the electromagnetic filed without reflection, with flat surface reflection, and with concave surface reflection. The detection area is a rectangle with 200 mm in length and 88 mm in width. The wave source is placed in the center while the morphing surface is placed at the top edge of the rectangle. d) Photographs of the QRF polyhedron arrayed fingertips gripping and lifting the fragile targets. e) The gripping tolerance of the above targets using different fingertips. f) Photographs (left) and schematic (right) of three kinds of robotic soles. g) The slope climbing experiments showing that only the QRF polyhedron array without gaps can support the robot to climb the steep slope.

For example, thanks to the zero‐Poisson's ratio property, the QRF polyhedrons can be arrayed as a morphing surface without gaps, which can serve as a dynamic mirror to modulate planar electromagnetic waves (**Figure** **6**a). The units of the morphing surface are covered with copper foil to create a tessellated reflective mirror and the shape can be adjusted to flat surface or concave surface (Figure 6b). When this mirror is placed around an electromagnetic wave source, the spatial electromagnetic field will change due to the superposition of the reflected wave and the source wave. And different superposition fields can be obtained by changing the shape of the mirror (Figure 6c; Figure S9, Supporting Information).

Additionally, to show the effect of anisotropy, a three‐by‐three arrayed morphing surface, in which a shape‐morphing unit comprises a QRF polyhedron and a twist & coiled polymer (TCP) actuator,^[^ ^45^ ^]^ was affixed to commercially available grippers to create a fingertip. We conducted a test using an xMate 3 Pro robotic arm and a Robotiq 2F‐140 gripper to assess the ability of the QRF polyhedron arrayed fingertips to handle fragile objects (Figure S10, Supporting Information). The low normal stiffness of the QRF polyhedron allows it to conform to the surface of the fragile object without causing any damage, while the high shear stiffness ensures sufficient lifting force on the target object. The results indicate that the QRF polyhedron arrayed morphing surfaces can securely hold a variety of fragile objects without leaving any visible scars on the surface (Figure 6d; Video S1, Supporting Information). To compare with the silicone blocks or arrayed silicone fingertips, we introduced an evaluation indicator called “gripping tolerance” to quantify the ability of a morphing surface to grip fragile objects safely (see Note S2, Supporting Information for details). Through a series of pick‐up experiments, we calculated the gripping tolerances of different fingertips with various fragile objects (Figure 6e; Figure S11, Supporting Information). Although both the silicone array and silicone block exhibit very high normal flexibility, the low shear stiffness obliges them to apply a larger compressive force to ensure sufficient lifting force, which usually results in damage to the objects. As a result, the gripping tolerances of the QRF polyhedron array are typically higher, except when handling objects with high water content, such as grapes and persimmons. In these cases, the lower normal stiffness of the silicone block provides a slight advantage.

Finally, we equipped a quadruped robot with QRF polyhedron arrays as the soles for climbing a steep slope with scattered rocks (Figure S12, Supporting Information). We compared three types of soles in the climbing experiments, including the QRF polyhedron arrays with gaps, without gaps, and an equal‐sized silicone block. It was anticipated that the units in the QRF polyhedron array with gaps would bend due to a lack of support from neighboring units, while the silicone block with low shear stiffness would slide down the slope. Only the proposed QRF polyhedron array without gaps could support the robot in climbing the steep slope (Figure 6f), and the experimental results confirm this hypothesis (Figure S13a; Video S2, Supporting Information). The robot equipped with QRF polyhedron arrays with gaps could oscillate up and down halfway but could not reach the top. In contrast, the robot with silicone soles failed to move forward and even slid down the slope due to gravity, highlighting the importance of zero‐Poisson's ratio and notable anisotropy (Figure 6g; Figure S13b and Video S3, Supporting Information).

## 3 Conclusion

In this research, we have proposed a QRF rate to quantify the extent to which a NRF structure approximates a rigid‐foldable one and accordingly designed and optimized a QRF polyhedron with notable anisotropy and zero‐Poisson's ratio. By arraying the QRF polyhedrons under different rules, we developed morphing surfaces for modulating planar electromagnetic waves, gripping fragile objects, or climbing steep slopes. The notable anisotropy ensures that the morphing surfaces maintain low normal stiffness while resisting large tangential forces. We then demonstrated its necessity in the object‐gripping and slope‐climbing experiments. Moreover, the zero‐Poisson's ratio ensures the close‐packing of the units, and this property is indispensable in both the electromagnetic wave modulation and climbing experiment. Compared to other foldable polyhedrons, only the proposed QRF polyhedron possesses both essential properties, and its anisotropy is outstanding.

Nevertheless, since the crease material is much softer than the faceted material, numerous slits appear on the interface when manufactured through 3D printing, limiting the lifespan of the arrayed surfaces. Therefore, new manufacturing technologies that can eliminate these slits will be beneficial in further improving the performance of the QRF polyhedron.

## 4 Experimental Section

### Fabrication of the QRF Polyhedron and Other Foldable Polyhedrons

The QRF polyhedrons and other foldable polyhedrons used in the experiments were designed in SolidWorks and then fabricated by a 3D printer (Stratasys, J750TM). The crease material was Agilus30 and the faceted material was a mixture of Agilus30 and VeroWhite to a shore hardness of 95.

### Mechanical Test

Compressive tests were performed on a custom displacement‐force test station, equipped with a 100 N force meter (Mavin, NA1) and a servo motor‐actuated moving stage (Figure S4a,Supporting Information). Each specimen was positioned on the stage and then moved at a rate of 0.01mm s^−1^. Shear tests were also conducted on the aforementioned test station. Specimens were placed horizontally and their two end surfaces were adhered to 3D printed fixtures (Figure 4d). The moving stage applies a shearing force to the specimens by driving the fixtures.

### Electromagnetic Waves Modulating Experiment

In the electromagnetic waves modulating experiment, the reflecting surface was composed of 14 units, each of which was fabricated by attaching a TCP actuator to the QRF polyhedron and adhering a copper foil to the external surface. Driven by the TCP actuators, each QRF polyhedron was folded to different heights to create differently shaped reflecting surfaces.

### Fragile Objects Gripping Experiment

In the fragile objects gripping experiment, three types of fingertips were utilized. The 3 × 3 QRF polyhedron arrayed fingertip was assembled by attaching 9 units to a 3D printed chassis. Each unit, measuring 12 mm in height and 20 mm in width, consisted of a QRF polyhedron and a TCP actuator. One end of the actuator was screwed onto the end surface of the QRF polyhedron, while the other end was attached to the chassis. The silicone block fingertip was fabricated by attaching a silicone block (Ecoflex 00–10, purchased from Smooth‐On company) of the same size as the QRF polyhedron array to the chassis. And the silicone arrayed fingertip was created by cutting the silicone block into a 3 × 3 array. For each experiment, two identical fingertips were affixed to a Robotiq 2F‐140 gripper, which was attached to an xMate 3 Pro robotic arm, to evaluate the ability to handle fragile objects. The distance between the two fingertips was gradually reduced, and the distances at which the objects were just lifted and just damaged were recorded to calculate the gripping tolerance.

### Slope Climbing Experiment

In the slope climbing experiment, a custom quadruped robot and a slope with a 30° inclination were used. Three types of soles were tested, a QRF polyhedron array with or without gaps and an equal‐sized silicone block. Each leg of the quadruped robot has 3 DOFs (Figure S12a, Supporting Information). The first joint moved the sole forward by actuating a four‐link mechanism, while the other two joints ensured the lifting and dropping of the sole. The slope was fabricated by cutting some arrayed square holes in a piece of acrylic board, and two kinds of 3D printed cubes were randomly placed in holes to simulate scattered rocks (Figure S12b, Supporting Information). The soles were the right size to cover 9 holes or cube bumps. Both the 3 × 3 QRF polyhedron arrays with and without gaps were developed by attaching 9 double‐layer QRF polyhedrons to a 3D printed chassis. And the silicone block sole was fabricated by attaching a silicone block (Ecoflex 00–10, purchased from Smooth‐On company) of the same size as the QRF polyhedron arrays to the chassis.

## Conflict of Interest

The authors declare no conflict of interest.

## Supporting information

Supporting Information

Supplemental Video 1

Supplemental Video 2

Supplemental Video 3

## Data Availability

The data that support the findings of this study are available from the corresponding author upon reasonable request.
