# Supplementary material for: Origami Morphing Surfaces with Arrayed Quasi‐Rigid‐Foldable Polyhedrons
Source: Adv Sci (Weinh). 2024 Jul 31;11(36):2402128. doi: 10.1002/advs.202402128 (PMC11422804; doi:10.1002/advs.202402128)
Supplement: Supplementary file 1 — Supporting Information [file ADVS-11-2402128-s004.pdf]

ADVANCED SCIENCE
Open Access

# Supporting Information

for *Adv. Sci.*, DOI 10.1002/advs.202402128

Origami Morphing Surfaces with Arrayed Quasi-Rigid-Foldable Polyhedrons

*Jiacong Li, Jiali Bao, Chengyeh Ho, Shuguang Li* and Jing Xu**

# Supporting Information

## Origami Morphing Surfaces with Arrayed Quasi-Rigid-Foldable Polyhedrons

*Jiacong Li, Jiali Bao, Chengyeh Ho, Shuguang Li*, and Jing Xu**

### Supplementary Note 1

### Mechanical model of the QRF polyhedron and experimental validation

The QRF polyhedron is composed of two types of materials: the crease material with a shore hardness of 30 and the faceted material with a shore hardness of 95. Given that the crease material is much softer, we assume that the deformation only occurs at the creases, a common simplifying method used in rigid-foldable polyhedrons. [18,19] Furthermore, for non-rigid-foldable polyhedrons, reducing the model into a truss structure also provides a reliable prediction of the folding behavior. [17,20] However, the deformation along the axis of the creases can be neglected due to the high QRF rate of the QRF polyhedron. Consequently, in this work it is assumed that creases undergo uniaxial extension along the direction $\vec{p}$ (**Figure S1**).

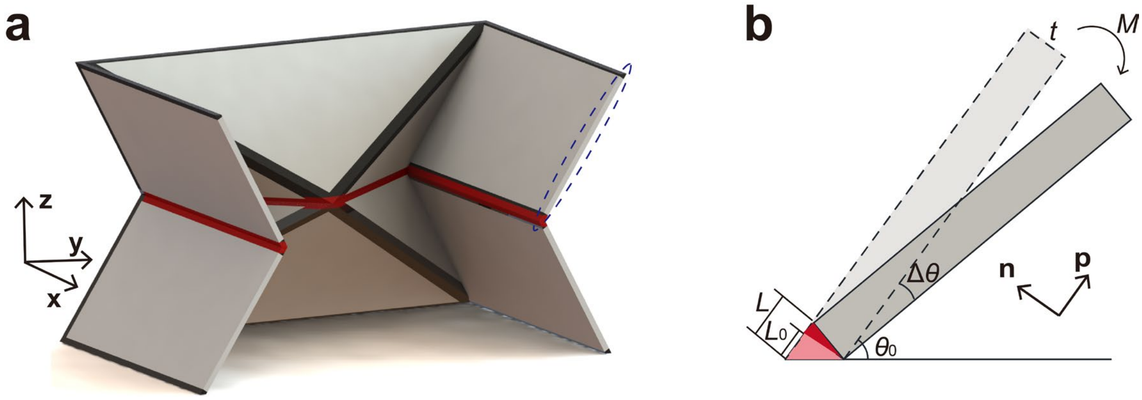


**Figure S1**. Schematic of the QRF polyhedron (a) and the deformation in the cross-section ZY-plane (b) during folding. Assuming that a rigid facet with thickness t undergoes an angular displacement of Δθ along the axial direction of the crease, the deformed cross-section is approximated by a sector created by the stretch (red) and a triangle that remains undeformed (pink).

Stretch ratio λ is more preferred to strain to depict the deformation for hyperelastic materials, which is defined as

$$\lambda = \frac{L}{L_0} \tag{S1}$$

where $L$ is the length after deformation, and $L_0$ is the initial length. In the pink triangle area, $L_0$ can be expressed as

$$L_0 = t\cot\theta_0 \tag{S2}$$

where $t$ is the thickness of the facet and $\theta_0$ is the initial tilt angle. Suppose the red stretched area is a circular sector, $L$ can be expressed as

$$L = L_0 + t\Delta\theta \tag{S3}$$

Combining Eqns. S1 to S3, we have

$$\lambda = \frac{\Delta\theta}{\cot\theta_0} + 1 \tag{S4}$$

After determining the stretch ratio of each crease, we need to establish the relationship between deformation and force. Hyperelastic materials exhibit non-linear elasticity under large strain, [45] thus a more complex model of the stress-strain relationship is needed instead of merely a linear coefficient such as Young's modulus. There are various hyperelastic models such as Gent, neo-Hookean, and the classical Mooney-Rivlin. Gent is reported to be a more accurate one among others,[16] while also offering mathematical simplicity. In addition, Gent being originally a phenomenological model is later proved to be related to the underlying microscopic structure of polymers.[16] Hyperelastic models mainly differ from each other in terms of the strain energy density function $W$. For the Gent model, the strain energy density function is

$$W^{\mathrm{Gent}} = -\frac{\mu J_\mathrm{m}}{2}\ln\left(1 - \frac{I_1 - 3}{J_\mathrm{m}}\right) \tag{S5}$$

where $\mu$ is the shear modulus, $J_\mathrm{m}$ is a coefficient related to the limiting chain extensibility of the material,[16] and $I_1$ is the strain invariant of the Cauchy-Green tensor, defined as

$$I_1 = \mathrm{tr}(B) = {\lambda_1}^2 + {\lambda_2}^2 + {\lambda_3}^2 \tag{S6}$$

where $B$ is the left Cauchy-Green tensor and $\lambda_i$ represents the principal stretches. Under certain assumptions, the engineering stress $\sigma$ based on the Gent model can be deduced in terms of the stretch ratio $\lambda$ which coincides with one of the principal stretches $\lambda_i$.[46] The engineering stress $\sigma$ and the tensile force $F_\mathrm{T}$ are

$$\sigma = \left(\lambda^2 - \frac{1}{\lambda}\right)\left(\frac{\mu J_\mathrm{m}}{J_\mathrm{m} - I_1 + 3}\right) \tag{S7}$$

$$F_\mathrm{T}(\mu, J_\mathrm{m}, h) = \sigma \cdot A \tag{S8}$$

where $A$ is the area normal to $\vec{p}$, and $h$ is half of the height of the QRF polyhedron during the folding process.

Now suppose the work done to compress the QRF polyhedron is directly transferred into deformation in the creases. Using the principle of virtual work, the relationship between the

compressive force $F$ and the bending moment $M$ on each crease is established as

$$F \cdot \delta h = \sum M \cdot \delta\theta \tag{S9}$$

where $\delta\theta$ is the associated variation of the angle of a crease to the virtual displacement $\delta h$. According to previous work, [18,19] the bending moment can be expressed as

$$M = \frac{F_{\mathrm{T}}(\mu, J_{\mathrm{m}}, h) \cdot \delta L}{\delta\theta} \tag{S10}$$

Inserting Eqns. S3 to S10, we have

$$M(\mu, J_{\mathrm{m}}, h) = F_{\mathrm{T}}(\mu, J_{\mathrm{m}}, h) \cdot t \tag{S11}$$

Combining Eqns. S9 and S11, we can calculat the force required for a certain displacement. It is worth mentioning that this is a generalized method and can be applied to any rigid- or quasi-rigid-foldable structures with hyperelastic creases.

To experimentally validate our model, we conducted compressive tests on 3D printed QRF polyhedrons. The creases were printed using Agilus30 while the facets using a mixture of Agilus30 and VeroWhite. Our proposed model and the previous linear model were used to fit the experimental data (**Figure 4a, S2 and Table S1**),[19] demonstrating that our model aligns more closely with the experimental results.

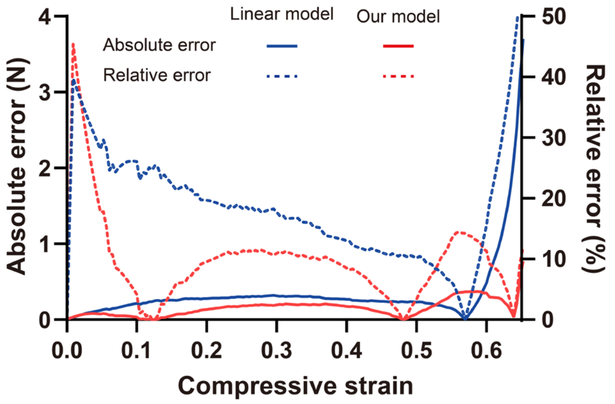


**Figure S2**. Errors between the two models and the experimental data.

**Table S1**. Fitted Parameters and the RMSE of Different Models

| Models | Our | | Linear |
|---|---|---|---|
| Parameters | $\mu$ | $J_m$ | $k$ |
| | 0.7 | 21.6 | 2.6 |
| RMSE | 0.19 | | 0.60 |

**Supplementary Note 2**

**Analysis of the gripping tolerance**

As shown in **Figure S3**, when a gripper is used to grasp an object, the compressive force increases with the displacement of the gripper after it makes contact with the object, until the displacement reaches the size of the object ($L_d$). If the displacement is too small, the compressive force is insufficient to generate enough friction to lift the object, while an overly large displacement can result in excessive compressive force, potentially damaging the object. We define the displacement that just manages to lift the object as $d_{min}$ and the displacement that risks breaking the object as $d_{max}$. Based on these definitions, the gripping tolerance ($T$) is defined as

$$T = \frac{d_{max} - d_{min}}{L_d - d_{min}} \tag{S12}$$

The larger the $T$, the greater the likelihood that the gripper can lift the object without causing damage. In other words, $T$ indicates the robustness of a gripper when subjected to simple position control.

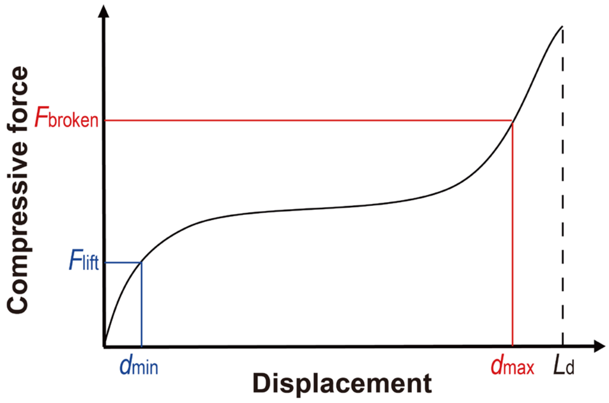


**Figure S3**. Schematic of the definition of $d_{min}$ and $d_{max}$.

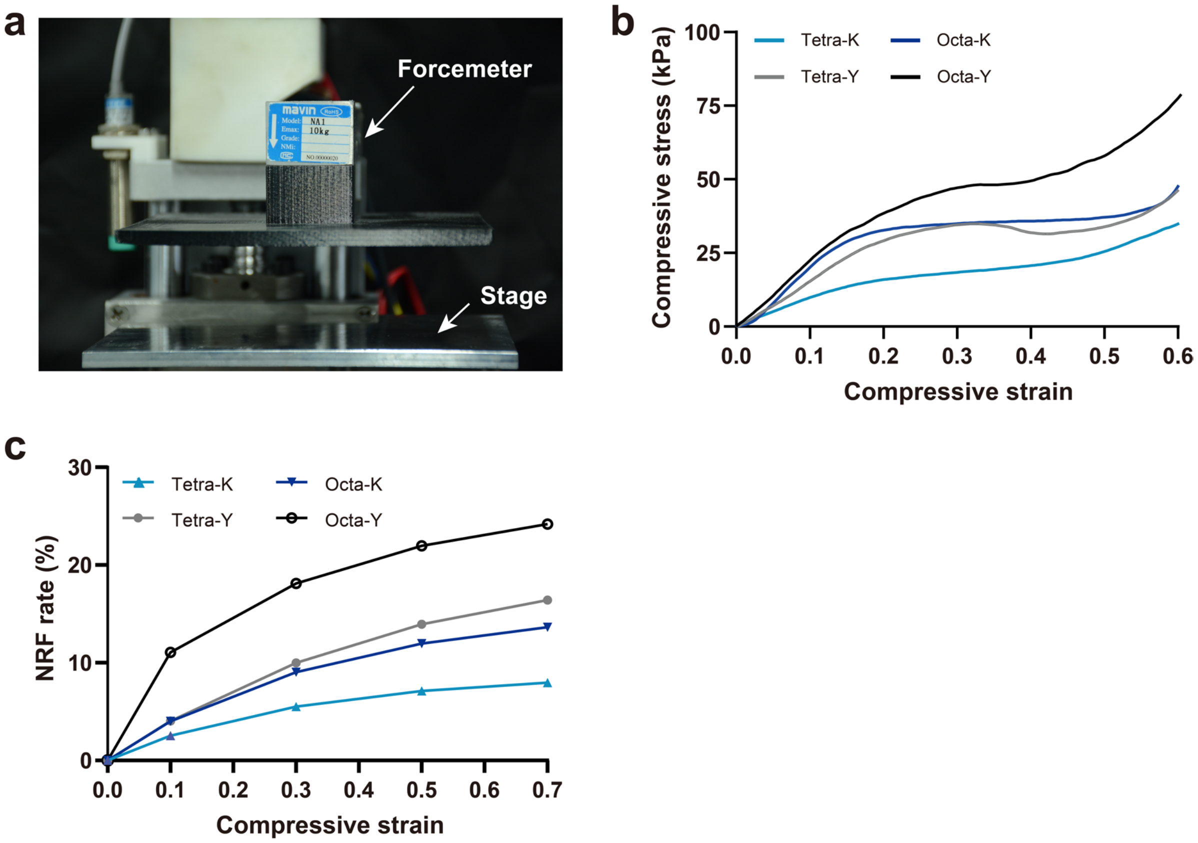


**Figure S4**. Compressive tests on different foldable polyhedrons and the setup. a) The setup was composed by a forcemeter and a motor-driven stage. b) Representative compressive stress-strain curves of the foldable polyhedrons. c) NRF rate-strain curves of the foldable polyhedrons.

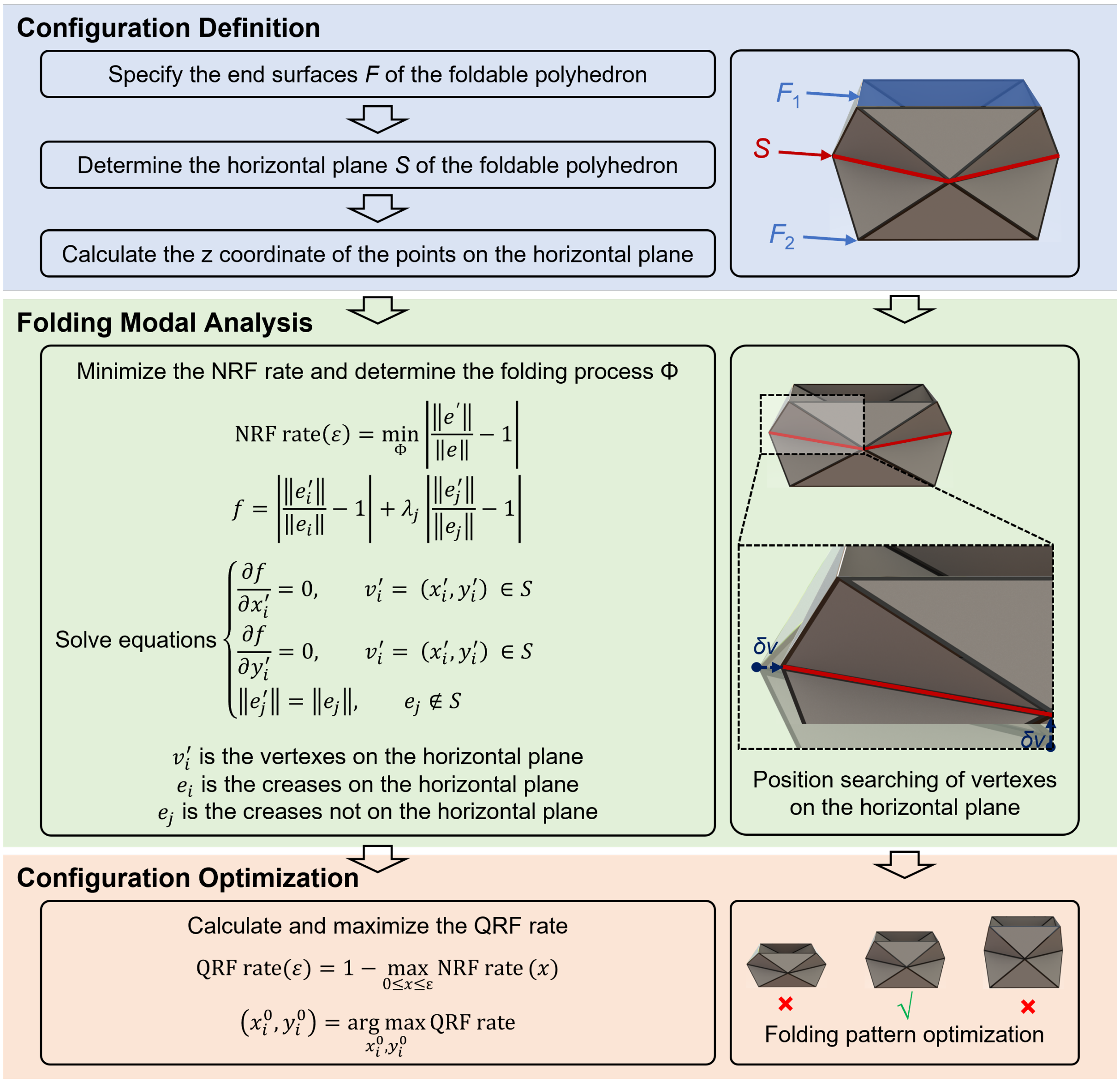


**Figure S5**. Optimization framework based on the QRF rate. The core process of the optimization framework is to minimize the NRF rate. The independent variable of the optimization is the coordinate values of each point on the horizontal plane when the height of the foldable cylinder is $h_{\text{hold}}$. The result is the functional relationship between all point coordinates and height $h_{\text{hold}}$, which represents the folding process $\Phi$. Simultaneously, we obtain the NRF rate and QRF rate under the initial configuration of the pattern, which is determined by the initial coordinate values of each point on the horizontal plane. Then, the gradient of QRF rate is calculated according to the partial derivative, and gradient descent is used to calculate the greatest QRF rate and corresponding coordinate values of each point on the horizontal plane, which determines the best initial configuration.

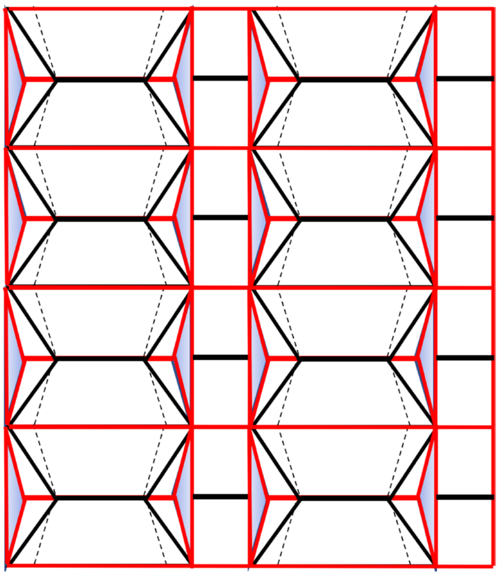

**Figure S6**. Pattern of the proposed QRF polyhedron.

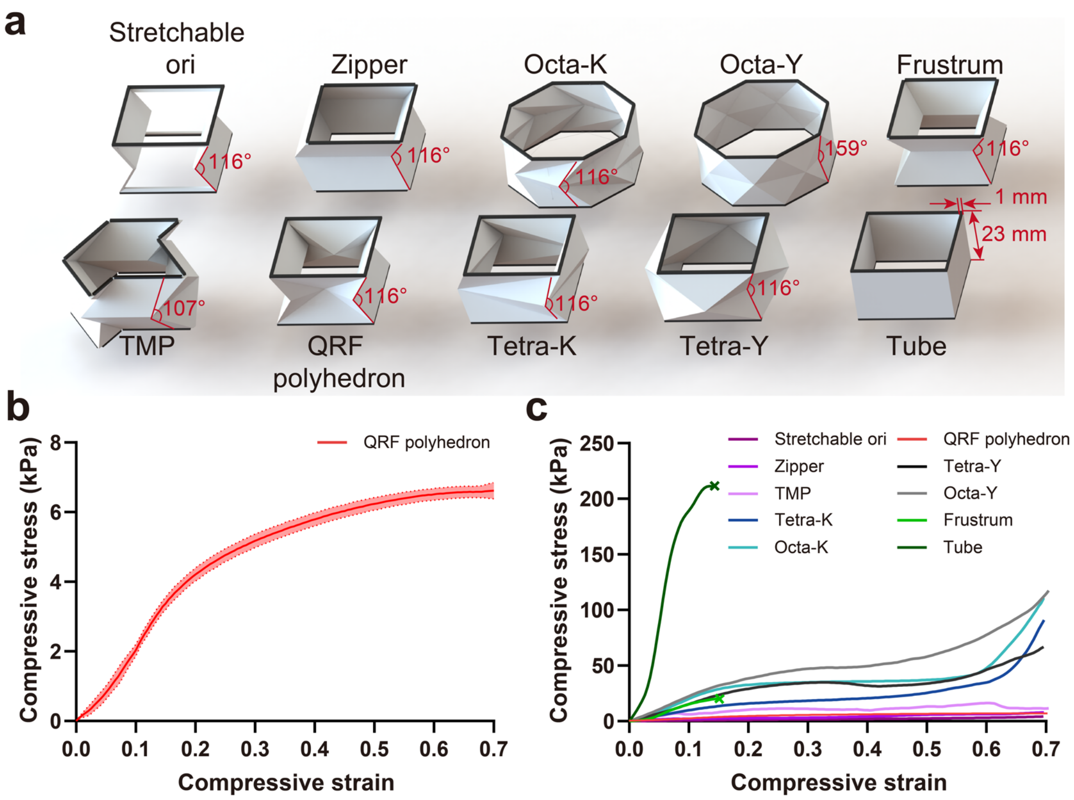


**Figure S7**. a) Geometric parameters for each of the foldable polyhedrons shown in **Figure 3e**, and the angle refers to the dihedral angle of the main crease. b) Compressive stress-strain curve of the QRF polyhedron. N = 3 technical replicates. c) Representative compressive stress-strain curves of the foldable polyhedrons.

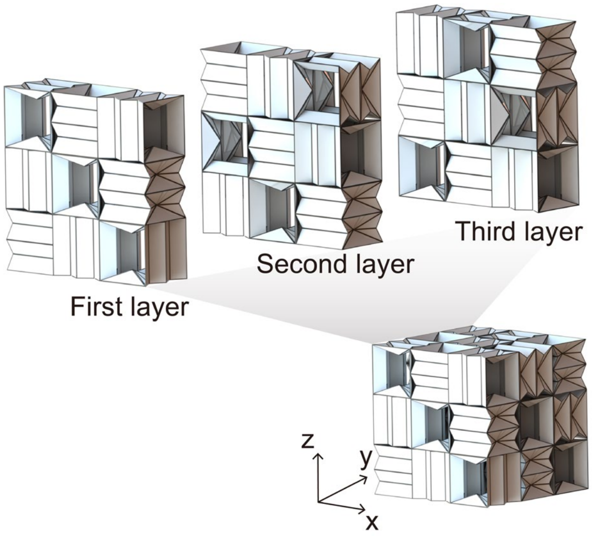


**Figure S8.** Schematic of the arrangement of units in the isotropic structure shown in **Figure 5a**.

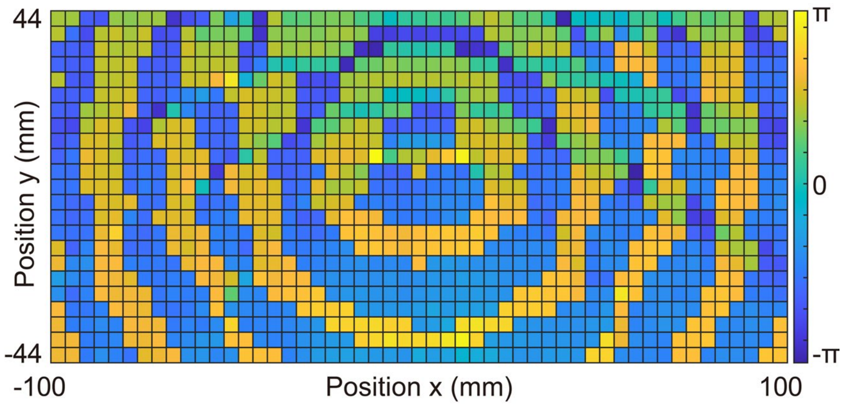


**Figure S9.** Phase difference of the electromagnetic filed between the flat surface reflection and concave surface reflection.

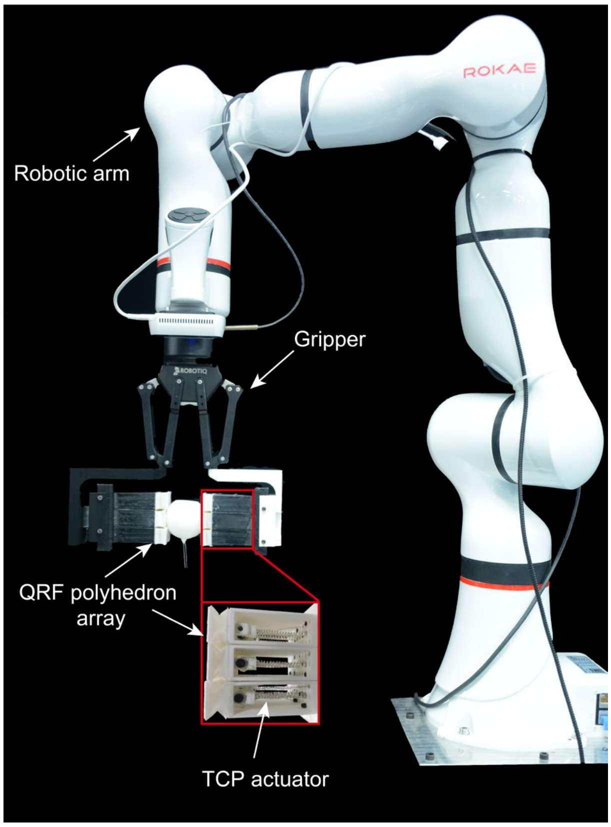


**Figure S10.** Setup of the fragile objects gripping experiment.

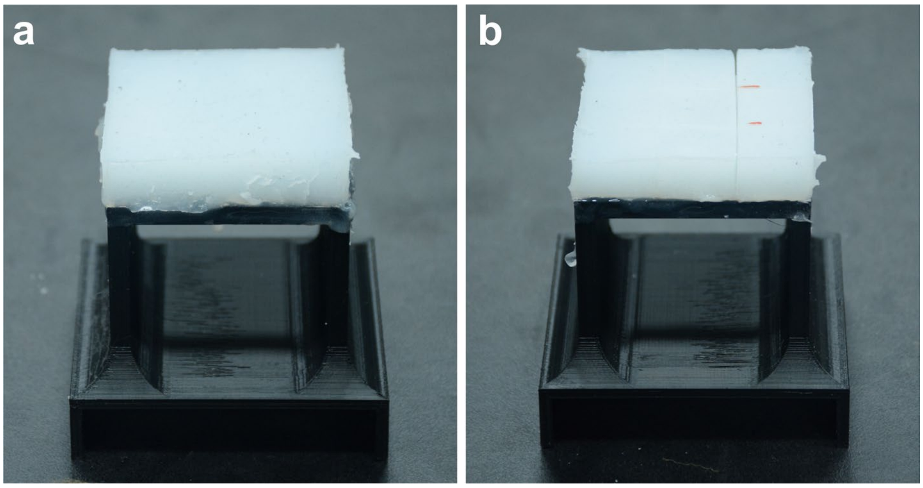


**Figure S11.** Photographs of the silicone block fingertip (a) and the silicone arrayed fingertip (b).

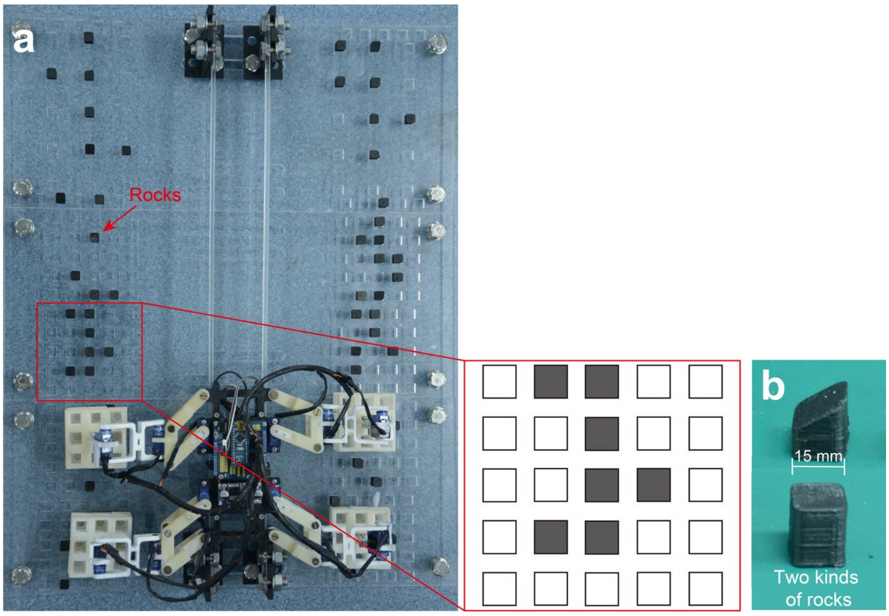


**Figure S12.** Slope climbing experiment. a) Photograph of the experiment setup, including a slope, a quadruped robot and scattered rocks. b) Photographs of the two kinds of rocks used in the experiment.

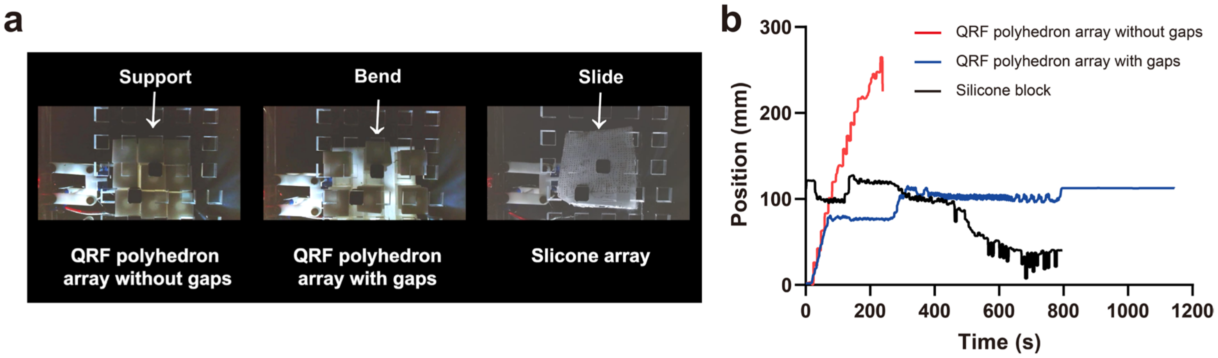


**Figure S13.** a) Photographs showing the deformation of three kind of soles in the slope climbing experiment. b) Representative position-time curves of quadruped robots with different soles.

**Supplementary References**

[46] C. O. Horgan, G. Saccomandi, *Rubber Chemistry and Technology* **2006**, *79* (1), 152.
